# Advances in Electrospun Materials and Methods for Li-Ion Batteries

**DOI:** 10.3390/polym15071622

**Published:** 2023-03-24

**Authors:** Sri Harini Senthilkumar, Brindha Ramasubramanian, Rayavarapu Prasada Rao, Vijila Chellappan, Seeram Ramakrishna

**Affiliations:** 1 Centre for Nanofibers and Nanotechnology, Department of Mechanical Engineering, National University of Singapore, Singapore 117576, Singapore; 2 Institute of Materials Research and Engineering (IMRE), Agency for Science, Technology and Research (A*STAR), #08-03, 2 Fusionopolis Way, Innovis, Singapore 138634, Singapore

**Keywords:** electrodes, separators, crosslinked nanofibers, needle-less, coulombic efficiency

## Abstract

Electronic devices commonly use rechargeable Li-ion batteries due to their potency, manufacturing effectiveness, and affordability. Electrospinning technology offers nanofibers with improved mechanical strength, quick ion transport, and ease of production, which makes it an attractive alternative to traditional methods. This review covers recent morphology-varied nanofibers and examines emerging nanofiber manufacturing methods and materials for battery tech advancement. The electrospinning technique can be used to generate nanofibers for battery separators, the electrodes with the advent of flame-resistant core-shell nanofibers. This review also identifies potential applications for recycled waste and biomass materials to increase the sustainability of the electrospinning process. Overall, this review provides insights into current developments in electrospinning for batteries and highlights the commercialization potential of the field.

## 1. Introduction

As traditional fossil fuel energy sources become increasingly scarce, it is important to explore alternative renewable resources. However, the intermittent nature of renewable energy means that efficient energy storage solutions are crucial. Using batteries is a practical way to reduce our dependence on fossil fuels and make the most of intermediate renewable energy sources. Batteries typically comprise two electrodes, the anode and cathode, separated by an electrolyte [1,2]. The transfer of Li ions between the electrodes is what generates the energy in batteries. The way in which energy is stored (through mechanisms such as Insertion/Intercalation, Conversion, and Alloying) has a direct impact on the amount of energy that can be stored. To increase capacity, a suitable nanostructure can be constructed as the active material for the electrode [3,4]. The design of nanostructures plays a critical role in providing a shorter ion pathway and effective electrical conduction, thanks to their larger surface area-to-volume ratio and superior mechanical strength. In particular, 1D nanostructures, such as nanorods, nanotubes, nanowires, and nanofibers, are often employed as electrodes to enhance capacity [5,6]. The use of 1D nanomaterials in batteries allows for greater flexibility in design, resulting in the development of more compact and efficient energy storage devices. The utilization of 1D nanowires as electrodes allows for a high density of active sites in a small area, leading to high energy densities, which is particularly advantageous in portable electronic devices where weight and size are critical factors.

**Figure 1:**
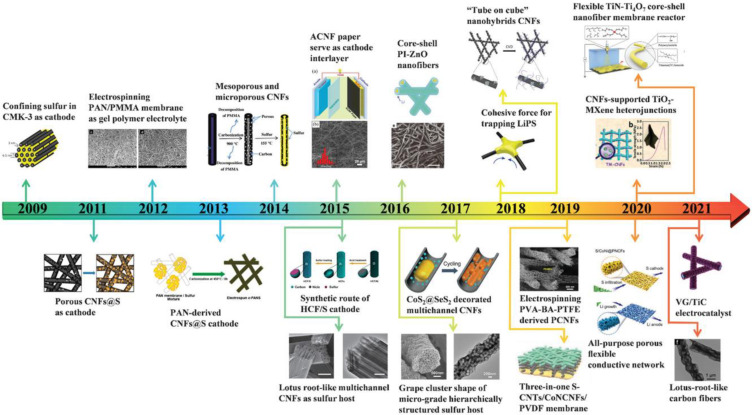
Timeline for electrospun nanofibers for the electrodes in LIBs (reused with copyrights from [16], Wiley, 2021).

The practicality and efficient production setup of nanofibers make them an ideal choice as the active material for LIB electrodes [7]. These nanofibers are highly flexible and can readily disperse active metals and metal oxides, which enhances the electrochemical performance. Figure 1 illustrates the timeline of electrospun material development for Li batteries [8,9]. There are several methods to prepare nanofibers, including wet chemical synthesis, self-assembly, vapor-phase approach, template-assisted synthesis, and hydrothermal synthesis [10,11]. However, electrospinning has gained significant attention due to its ease of large-scale production, affordability, and high efficiency [12,13,14,15].

**Figure 2:**
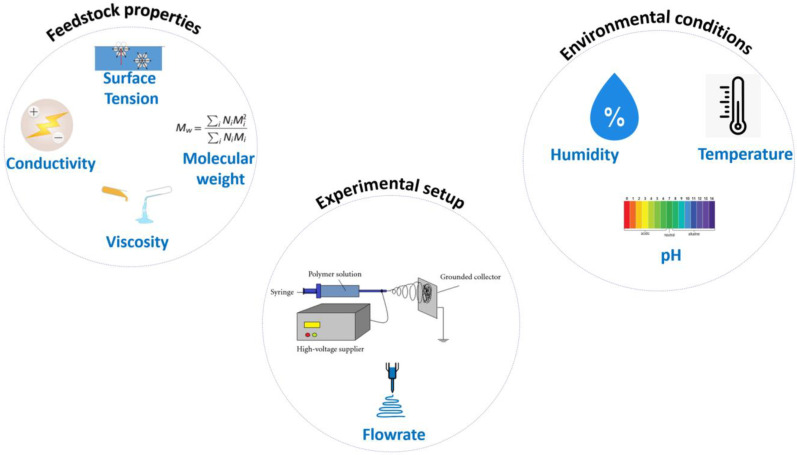
Factors affecting the Electrospinning process.

The underlying principle behind the electrospinning technique is based on leveraging the surface tension of the polymer and manipulating the electric forces to produce fibers with customizable properties [17,18]. Furthermore, the characteristics of the resulting fiber can be fine-tuned by altering properties, such as the solution viscosity, molecular weight of the precursor solution, applied voltage, humidity, flow rate, and the distance between the spinneret and collector, as demonstrated in Figure 2 [19]. Electrospun nanofibers are highly versatile and are employed in a broad spectrum of applications, spanning from filtration, drug delivery, sensors, energy storage, biomedical, and textiles [20,21]. The article defines the nanoscale as having at least one dimension below 100 nm. The fibers explored in the review were much smaller than traditional materials, so the authors named them “nanofibers”.

By making changes to the structure of nanofibers and adjusting the electrospinning process, it is feasible to produce nanofibers with specific properties. In the case of the nanofiber for the electrodes, direction-oriented electrospinning [22,23], in situ electrospinning [24], hybrid composite using single pot electrospinning, and fork-like electro-spun single crystalline cathodes [25] have been reported previously [21,22,23,24]. Nanofibers designed for use as battery separators can be produced more efficiently using a syringeless technique, which yields beadles fiber. Additionally, the electrospinning method can be utilized to create “smart” nanofibers, and the inclusion of flame retardants in the shell of core-shell nanofibers can aid in battery shutdown [26]. Additionally, the electrospinning method can be used to create nanofiber composites that are doped [27], encapsulated [28], porous [29], or bendable as well. Despite these advances, there are still limitations to the sustainability and efficiency of nanofibers in LIBs, prompting ongoing research for improvements [30]. In addition to reusing or recycling components, it may be worth exploring the extraction of raw materials from sustainable bioresources such as biomass. This is because the materials used to make LIBs are fleeting, and the current extraction processes are not environmentally friendly [31,32,33]. Consequently, the focus of this study is on the advancement in the materials and techniques used in the electrospinning process to solve the shortcoming and result in improvement in the performance of lithium-ion batteries for a sustainable future [34,35]. Researchers are always looking for new and improved electrospun electrode materials and techniques for high-performance rechargeable LIBs. The advancements that enable the use of electrospun nanofibers in batteries, which have not yet been well studied, are facilitated by this review in helping researchers think through those improvements in depth.

## 2. Advancement in Electrospun Cathode Materials

The positive electrode of an electrochemical cell, known as the cathode, plays a vital role in maintaining the potential window and specific capacity of the cell. As lithium is inherently unstable, composite cathodes are preferred over pristine lithium, as they offer high stability, a broad potential window, and adjustable redox potentials. Commonly used cathode materials include vanadium oxides, tri chalcogenides, iron compounds made up of oxides and phosphates, and transition metal oxides [36]. The choice of materials for synthesis must be wise since it affects how well the battery works. The raw material used must also be free of impurities because this impacts the safety and lifespan of the battery as well as its affordability [37]. There are two mechanisms used to store energy in the cathode component, one of which is the conversion type, which involves changing the cathode’s structure while transporting ions. Low electronic conduction and significant volume expansion, in general, limit the specific capacity. There is another reversible method by which the cathode structure serves as the host while ions are intercalated. Compared to chalcogens, the transition metal oxide and polyion structures are more commonly employed because of their reversibility during cycling [38].

**Figure 3:**
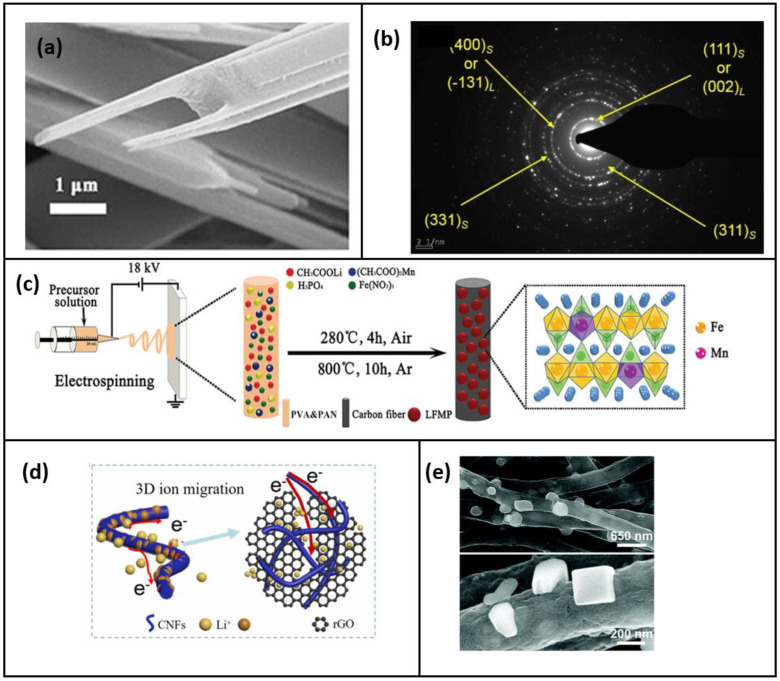
(**a**) TEM image of electro-spun fork-like K_2_V_8_O_21_ (Reprinted with permission from Ref. [25] 2018, Frontiers) (**b**) SAED patterns of the nanofiber showing the spinel (S) and layered (L) phase of LiM_2_O_4_ (Reprinted with permission from Ref. [42] 2020, Elsevier) (**c**) Schematic of synthesis of the LFMP/C nanofibers (**d**) Diagram showing r GO improving material conductivity and ion diffusion rate (Reprinted with permission from Ref. [47] 2023, Elsevier) (**e**) High-magnification SEM images showing the cubic LVP nanoparticles embedded in the CNF networks (Reprinted with permission from Ref. [52] 2019, RSC).

Among the layered structure available, LiCoO_2_ is widely used because of its high theoretical capacity of 442 mAh g^−1^, but its poor cyclic, structural, and thermal stability, as well as the limited availability of Co limits, is commercial viability [39,40]. As a result, other widely accessible materials, such as vanadate and vanadium oxide, are used as substitutes. Additionally, the reversibility and specific capacity significantly increase when K^+^ ions are introduced to the vanadium oxide layers because of the expanded interlamellar space and the pillar effect. In recent days, fork-like electro-spun K_2_V_8_O_21_ single crystalline cathode material was employed in a demonstration by Hao et al. [25]. Here, the recrystallization process after electrospinning transforms the nanofibers into a fork-like structure, observed in a scanning electron microscope (SEM), as presented in Figure 3a, and this structure has a larger contact area between the electrode and electrolyte, improving the lithium-ion diffusion. The fork-like structure has several benefits, including reduced volume expansion, efficient lithiation, and delithiation. The volume change is impeded because there is enough space between the two rods of the fork. The structure of the single-crystal material employed maximizes the efficiency of the lithiation and De lithiation cycle. The specific discharge capacity was 200.2 mAh g^−1^ at a current density of 50 mA g^−1^, and the retention capacity after 100 cycles was 96.52%.

Recently, colloidal titania nanoparticle (TiO_2_ NPs)-doped LiCoO_2_ nanofibers [41] were produced by increasing the calcination temperature in addition to changing the operating parameters, which improved stability, crystallinity and caused the binary phase (LiCoO_2_/TiO_2_) transformed into a ternary phase (Li_2_CoTi_3_O_8_/TiO_2_). The initial capacity retention increased from 65 to 90%, and the specific capacity increased from 78 to 148 mAh g^−1^ by raising the calcination temperature from 400 to 700 °C and doubling the molar ratio Li: Co (2:1), respectively. The ternary structure’s discharge capacity was 82 mAh g^−1^ at 0.1 C, while the binary LiCoO_2_ had a higher specific capacity. The ternary structure’s non-1-dimensional nanoparticles reduced the diffusion distance for the lithium insertion, which, in turn, decreased the discharge capacity. The degradation of fiber caused by higher calcination temperatures can result in the loss of the fiber network, which, in turn, can affect the electrochemical characteristics and reduce the overall strength.

The other material which is being explored is the spinel structure, with a LiM_2_O_4_ structure, and this structure has a high-rate capacity because the internal 3D structure improves Li diffusion. However, these materials often experience capacity fading. N.H. Vu et al. [42] have developed a composite anode as an improvement of the already existing composite by Kitajou et al. [43], which exhibited low average voltage and was fabricated using a solid-state synthesis method. In general, the spinel structure present in the composite cathode increases the capacity but reduces the average voltage. Thus, to strike a balance between these properties, the composite was fabricated to have an optimal ratio of the spinel phase Li_2_MnTiO_4+z_ (0.5LiMnTiO_4_; 0.5Li_2_Mn0_5_Ti0_5_O_3_) as shown in the SAED pattern in Figure 3b. At C/10 and C Ratings, the 1-D, hetero-atom cathode may produce capacities of up to 210 and 150 mAh g^−1^, respectively. However, as the cycling process continues, the layered structure transforms into the spinel structure, causing the cathode’s capacity to decline to 95.6% when measured at 1 C after 100 cycles. Thus, the in situ formation of spinel compound after several cycles of discharging must also be noted.

To overcome the capacity fading brought by the spinel structures discussed, the material is doped with the elements, such as Mg, Ni, Al, and Cr, which improves the electrochemical characteristics by lowering the Jahn–Teller effect. Using the in situ electrospinning technique, as shown in Figure 3c, Iso valent Mn^2+^ was doped in LiFePO_4_ (LFP). Because it occupies the Fe sites, this doping preserves the original crystal structure [44]. A Li (FeMn)PO_4_ (LFMP) solution is created because of a minute difference in the radii of the high spin Fe^2+^ and Mn^2+^ ions, which causes expansion and creates more room for the insertion of Li ions [45]. To achieve a uniform dispersion, the dopped composite was first made using the sol–gel method. Later, however, using the electrospinning procedure, a network of nanofiber material with evenly embedded nanoparticles was established. The coulombic efficiency of this doped LiFe_0.8_Mn_0.2_PO_4_/C nanofiber is 92.4%, a sizeable 169.9 mAh g^−1^ initial capacity, 160 mAh g^−1^ reversible capacity after 200 cycles at 0.1 C, and 93 mAh g^−1^ specific capacity at 5 C [24].

LiFePO_4_ and LiMnO_4_ are some of the well-established cathode materials. These polyionic compounds have adequate power capacity and remarkable structural stability, and their typical chemical structure is XO, where X represents P, Si, S, etc. [46]. By introducing carbon fibers to enhance ionic conductivity, fabricating porous structures to mitigate degradation, and coating nanofibers to increase capacity, it is possible to optimize the performance of electrospun nanofibers composed of polyions. Thus, according to Liu et al. [47], by introducing carbon fibers to enhance ionic conductivity, fabricating porous structures to mitigate degradation, and coating nanofibers to increase capacity, it is possible to optimize the performance of electrospun nanofibers composed of polyions. Ion mobility is increased because of the formation of many new ion migrating channels (8.82 10 12 cm^2^ s^−1^), as shown in Figure 3d. As a result, a discharge capacity of 167.1 mAh g^−1^, 118.9 mAh g^−1^, and long-cycle stability (98.9% of original capacity after 200 cycles at 1 C rate) were measured.

By utilizing ionic liquid-based electrospinning, it is possible to form a porous structure that can accommodate volume change, thus preventing the breakage of active material caused by increased mobility [48]. The technique involves the interaction of the liquid’s ions with the existing active particles, which modifies the surface property and leads to a uniform dispersion of the active materials in the precursor solution. Furthermore, the high ionic conductivity of the ionic liquid enhances the surface tension and conductivity, resulting in the formation of a porous structure [49,50]. A lithium vanadium phosphate Li_3_V_2_(PO_4_)_3_ (LVP)-cathode was created using this ground-breaking method, with the LVP of the Nanocube dimension embedded in the N-doped CNF, as shown in Figure 3e. The precursor made of PVA and LVP is not very stable in general, so methods, such as creating an organic electrospinning precursor solution or coating LVP nanoparticles into a network of preformed nanofibers, may help to improve the stability and spinnability and, thus, the properties of the fabricated fiber [51]. The ionic liquid 1-n-butyl-3-methylimidazolium dihydrogen phosphate ([Bmim]H_2_PO_4_) was introduced for the first time in this study by Yi Peng et al. [52], and in addition to aiding the generation of a stable precursor, it also acts as a structure-directing agent. Additionally, this acts as a carbon supply for the in situ carbon coating layer that is nitrogen-doped. The constructed LVP-NC/NCNF that results from these experiments has a high discharge capacity of 143.8 mAh g^−1^ at 1 C over a long cycling life of over 1750 cycles and can even obtain a high C-rate capacity of 143.6 mAh g^−1^ at 5 C after 1000 cycles.

**Figure 4:**
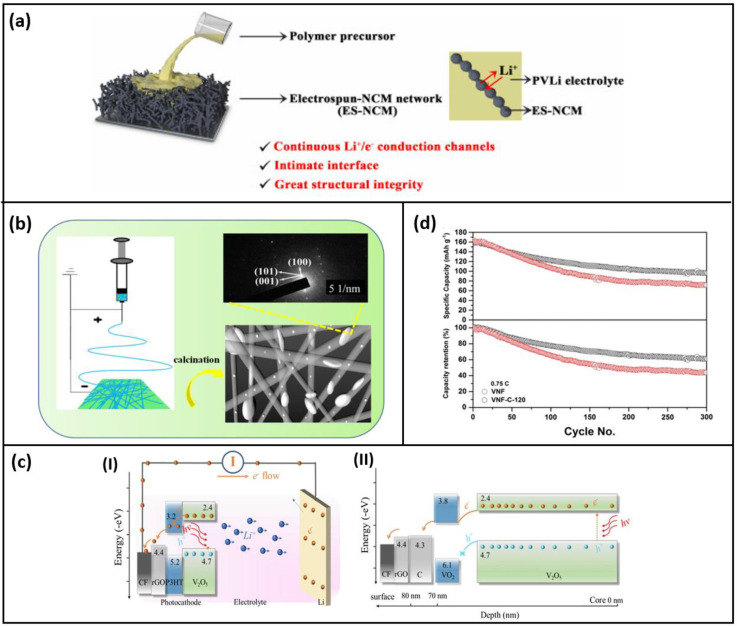
(**a**) Schematic illustration for the construction of composite ES–NCM cathode based on proposed interface engineering strategy (reused with copyrights from Ref. [53] 2022, Elsevier) (**b**) Schematic illustration of the free-standing (010)-oriented LiFePO4/carbon fibers (reused with copyrights from Ref. [23] 2020, Elsevier) (**c**) Schematic representing the photo charging mechanism of (I) VNF and (II) VNF-C-120 (reused with copyrights from Ref. [54] 2022, Wiley) (**d**) Cycling stability and capacity retention tests at 0.75 C of VNF (red) and VNF-C-120 (gray) (reused with copyrights from Ref. [54] 2022, Wiley).

A polymer with improved interfacial contact and assisting Li^+^/e^−^ transport is produced by infiltrating Li^+^ conductive polymer electrolyte precursor into the electro-spun LiNi_0.5_Co_0.2_Mn_0.3_O_2_ (ES-NCM) [53] network and then polymerizing in place, as shown in Figure 4a. Ionic liquid, as well as plastic–crystal electrolytes, can be used in addition to the liquid electrolyte polymer (PVLi) added as the Li Conductive additive. During successive Li^+^ insertion/extraction, the infiltrated ductile PVLi electrolyte would aid in decentralizing the mechanical stresses of the inner cathode particles. The battery fabricated with this composite as a cathode has a capacity retention of 74.0% after 80 cycles and a discharge capacity of 131 mAh g^−1^ at 0.1 C. High-energy-density lithium batteries make use of this cathode material.

Polyvinylpyrrolidone (PVP) was used as the regulator in a single-pot electrospinning procedure to create a hybrid LiFePO_4_/carbon nanofiber by orienting the LiFePO_4_ crystals to grow in the (010) face plane [23]. Figure 4b, By using crystal engineering, it could be possible to create improved lithium storage and a reliable Li transfer channel. The samples were prepared via this method without the use of polymeric binders by adjusting variables, such as the PVP content in the precursor solution, the heat treatment temperature, and the ambient humidity. After 500 cycles, the manufactured fiber showed 98.2% retention capacity at 0.5 C, 152 mAh g^−1^, discharge capacity, and 108.6 mAh g^−1^ good rate performance at 5 C. The author noted that this technique might also be utilized to create orientated crystal LiMPO_4_/carbon nanofiber hybrids as well. Thus, the electrospinning technique’s flexibility is demonstrated by the prospect of crystal engineering.

In addition to producing electrodes for electrochemical batteries, electrospinning is also used to construct the cathode for photo-responsive batteries, and the working is given in Figure 4c. Vanadium oxide nanofibers coated with conductive carbon, such as the VNF-C-120 [54], are used as the cathode for lithium-ion batteries that can be recharged using sunlight. Given its photo responsiveness, reversible redox active behavior, necessary bandgap, and affordability, V_2_O_5_ is selected as the ideal cathode. Improved surface chemistry, ion transport, and electrical conductivity are the goals of carbon coating. Additionally, it keeps the electrode from coming into direct contact with the electrolyte and accommodates the volume change that occurs during continuous cycling [53]; also, the cyclic stability is increased considerably after 300 cycles post-carbon coating, as shown in Figure 4d.

Table 1 provides an overview of the recent advanced electrospun cathode material.

## 3. Advancement in Electro Spun Separator Materials

A crucial component of LIBs is the separator, which is situated between the positive and negative electrodes. It permits the movement of ions between the electrodes while strictly prohibiting the flow of electrons, as electron transfer can lead to a short circuit. The primary focus of separator innovation is improving safety conditions since the separator does not undergo any electrochemical reactions [55]. Initially, a liquid electrolyte was employed as a separator, which consists of appropriate metal salts dissolved in aqueous or non-aqueous solutions. Later, the solid polymer electrolyte (SPE) was developed to address the electrochemical instability problem encountered by the traditional liquid electrolyte. In spite of the high conductivity of the liquid electrolyte, they are prone to leakage, are flammable, and readily corrosive, making them unsuitable for applications, primarily due to safety concerns [56,57].

The use of polymer as an electrolyte was problematic due to its inadequate interfacial compatibility with electrodes and poor ionic conductivity. To overcome this, a functional polymer electrolyte was developed, where modifications were made to the polymer host, inorganic filler, metal salt, and liquid solvent. Here, the presence of a flexible polymer chain and the absence of crystallinity facilitated enhanced ionic diffusion; however, the desired ionic conductivity was not achieved. To address this, solid polymer electrolyte (SPE) was developed using polyolefins such as polyether, polysulfone, polysiloxane, and polyborate as the polymer matrix [58,59,60]. Later, plasticizers were added to the solution to increase the ionic conductivity. This addition of plasticizers leads to swelling of the solution forming a viscous solution known as the gel polymer electrolyte (GPE). Polyethylene oxide (PEO), poly (methyl methacrylate) (PMMA), polyacrylonitrile (PAN), polyvinyl acetate (PVAc), and polyvinylidene fluoride (PVDF) were the generally used Polymer for GPE [61,62,63]. Another method to improve safety was through co-axial electrospinning, where inorganic ceramics are coated on polymers to further improve their mechanical properties and to provide thermal resistance [64,65]. However, with continuous cyclic performance, the coated ceramic nanoparticles self-aggregate and fall off, subduing the purpose of the coating [17,57].

**Figure 5:**
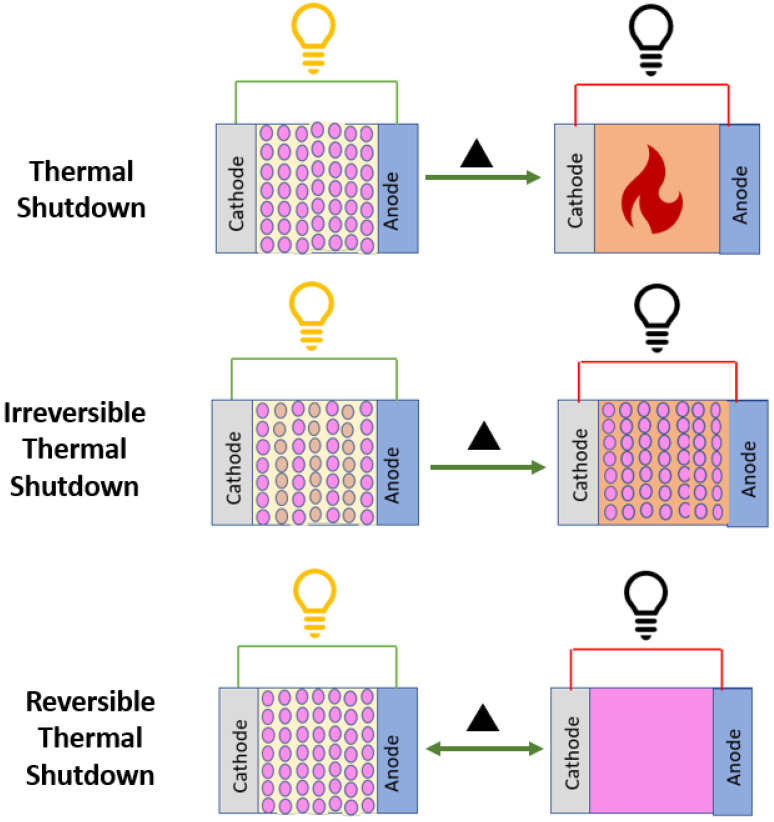
Schematic of thermal shutdown, irreversible thermal shutdown, and reversible thermal shutdown mechanism in energy storage systems.

Extreme safety-related problems, such as short circuits, lead to thermal runaway, and then a potential explosion takes place when separator shrinking happens [66] due to a significant increase in temperature within a battery. By utilizing bilayer or multilayer separators with polymers of varying thermal characteristics, the shutdown effect is achieved, which prevents the potential risk [67]. Here, one of the polymers with a lower melting temperature, melt and obstructs the other polymer’s pores, ceasing the electrochemical reaction and preventing any more electrochemical reactions in the cell. Similar to fuses in an electrical circuit, this multilayer separator is utilized in an electrochemical cell, as described in Figure 5. It is crucial to distinguish between reversible thermal shutdown, irreversible shutdown, and separator breakdown in energy storage systems. Reversible thermal shutdown is a temporary increase in internal resistance due to elevated temperatures, while irreversible shutdown leads to permanent damage. Separator breakdown occurs when the separator fails, causing an internal short circuit. Identifying these failures helps to prevent further damage and improve safety. Addressing reversible thermal shutdown requires improving cooling, while irreversible shutdown and separator breakdown may need significant design or operational changes. The widely commercially used layered composite is Polypropylene (PP) and Polyethylene (PE), the PE (Tg = −68 °C, Tm = 135 °C), which has a lower temperature melting point and blocks the pores of PP (Tg = −20 °C, Tm = 165 °C). Here, the temperature difference between the melting point and the shutdown window is small, leaving some possible implicit dangers in place. As a result, multi-layer materials with a large difference between their melting and shutdown points are favored for improved safety precautions. such as the Coaxial membrane and PEEK/PMMA/PEEK) [68] membrane.

**Figure 6:**
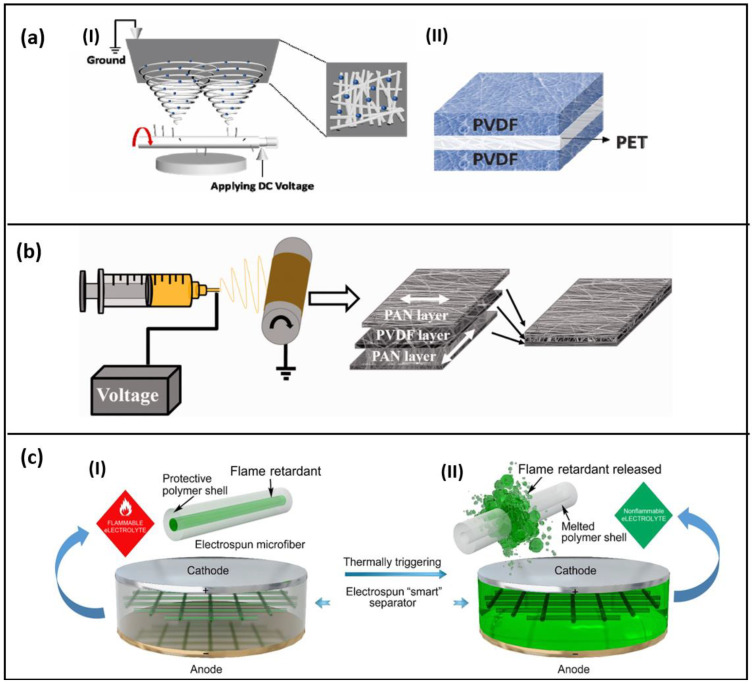
(**a**) Schematic of syringeless electrospinning of PVDF non-woven composite Web (I) Structure of nonwoven separator (II) (reused with copyrights from Ref. [69] 2022, Elsevier) (**b**) Fabrication of directionally oriented composite nanofibers (reused with copyrights from Ref. [22] 2022, Sage) (**c**) Smart electro-spun nanofibers with core-shell structure (I)-before thermally triggering (II)-after thermally triggering releasing flame retardants that suppress ignition. (reused with copyrights from Ref. [72] 2017, Science advances).

To achieve economic and mass production, syringeless electrospinning techniques were used to fabricate a PVDF composite non-woven web, as shown in Figure 6a [69,70]. When compared to conventional electrospinning, the production rate was eight times higher for syringeless spinning. Additionally, via this method, it was easier to add the colloidal inorganic fillers into the non-woven web as the issue of needle block during precipitation will not occur. Since the parameters involving the syringe are not added, it is easier to optimize the production process as well. Polycaprolactone (PCL) is a biocompatible polymer that can be electro-spun to synthesize the separator; these separators can sustain high discharge current and possess good ionic conductivity; however, the application is limited since the melting point is 60 °C and it cannot impede the formation of lithium dendrites [71]. A high-performance composite separator was fabricated using a high-speed roller orientation receiving method of electrospinning to obtain an oriented composite of 0°PAN/PVDF/90° PAN directions [22]. Figure 6b depicts the constructed triple-layer oriented sandwich composite. When reassembled into a battery unit, it provides a specific capacity of 165 mAh g^−1^ at 0.5 C, with a 90% capacity retention rate after 100 cycles. Tensile strength increased by two–three times in both the longitudinal and transverse axes.

Using polyimide (PI) as a matrix, poly (amic acid) (PAA) as a reinforced layer, and polyetherimide (PEI) as the organic functional layer, an advanced polyimide nanofiber was developed [73]. Due to the inadequate physical contact between the nanofibers, the Polyimide Electro spun Nanofiber alone has a loose structure and offers a chance of disintegration. As the evaluated Tensile strength was nearly 30 MPA higher than the non-modified PI Nanofiber, the reinforcing by additional processing resulted in an increase in the chain length of the PI backbone and in situ bonding that gives good mechanical characteristics. For 100 cycles of testing, the cyclic stability across 1 C and 5 C discharge rates was excellent. Non-woven separators of PE fabricated by other processes, such as the melt blown process, have a larger pore size which is not efficient for the shutdown process, but electrospinning leads to the synthesis of nanofibers of smaller diameter; however, in the synthesis process, the PE has poor solubility to the solvent; thus, more soluble Poly-1-butene Nanofiber was used for the electrospinning [74], and it has other properties similar to that of PE. This Polyethylene based separator was highly suitable as it possesses the required shutdown properties, and by adjusting the proportion of the polymers (monomer, copolymer) present, the melting point can be altered as well.

The occurrence of a battery shutdown due to high temperature is undesirable, especially with the advancements in circular economy practices, such as the second-life usage of batteries. If an irreversible separator is used and a thermal shutdown occurs, the entire battery unit will stop all electrochemical activity, resulting in a significant amount of waste which is not desirable given the current demand for raw materials. To address this issue, separators are being developed with a reversible shutdown mechanism using smart materials that can react to changes in environmental factors, such as heat, pH, concentration, and pressure, among others. This approach aims to prevent the entire unit from ceasing operation and potentially contribute to a more sustainable and efficient use of battery resources. Phase-change materials (PCM), thermoresponsive polymers, and shape memory polymers are the currently known advanced materials that offer a reversible shutdown mechanism for battery separators [75]. Microfibers were fabricated using an electrospinning technique, forming a core-shell structure, where the organophosphorus-based flame retardant, namely, the triphenyl phosphate (TPP), is chosen as the core, and the shell is made up of poly (vinylidene fluoride–hexafluoropropylene) (PVDF-HFP) [72]. This smart non-woven separator (TPP@PVDF-HFP) reduces the possible negative effects as the direct exposure of the flame retardant with the electrolyte is inhibited due to the encapsulation; the working of this smart electro-spun nanofiber is demonstrated in Figure 6c.

**Figure 7:**
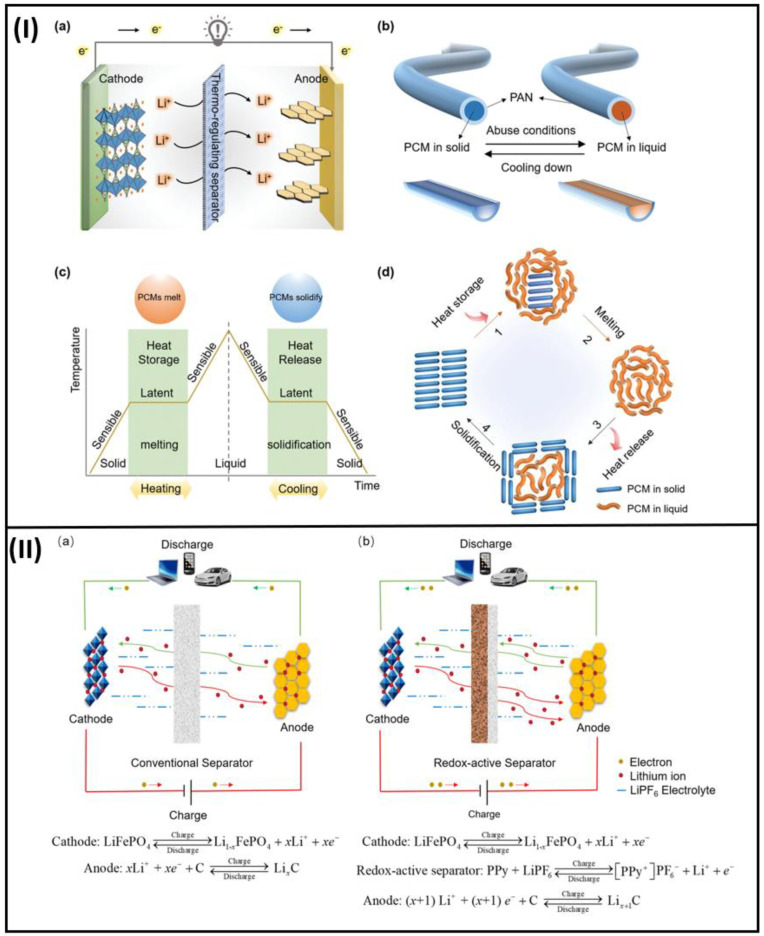
(**I**) An example of a schematic for a thermoregulating separator that lowers the internal temperature of LIBs under abusive situations (**a**) Design of the thermoregulating separator with LIBs. (**b**) The thermoregulating separator’s schematic arrangement. (**c**) The thermoregulating separator’s approach for regulating the interior temperature of LIBs (**d**) A change in the specific structure of PCMs because of heat absorption and heat release (reused with copyrights from Ref. [76] 2021, Wiley) (**II**) Working of LFP/Li Batteries (**a**) with Conventional Separator (**b**) with novel redox active Separator (reused with copyrights from Ref. [82] 2022, Elsevier).

Phase change material (Paraffin Wax) was enclosed in a polymer sheath of polyacrylonitrile nanofibers using a coaxial electrospinning approach by Xian Luo Hu et al. [76]. This bead-free nanofiber has outstanding electrolyte absorption and porosity because the fibers are well-linked, as shown in Figure 7I. The number of accessible routes for ion transportation increases with increasing porosity. The Separator Nanofiber’s thermoregulation and electrochemical performance were both enhanced by the addition of the PCM. Thermal conductivity is poor in PCM; thus, conductive substances, such as aluminum, copper, and nickel, are added as foam to the battery thermal management systems (BTMS) on the market to improve the thermal qualities. The polymer shell that would surround the PCM Core using the Co-Axial electrospinning approach might now additionally incorporate a thermally conductive substance for the separators. The materials used in BTMS, such as conductive copper foam, aluminum foam, carbon fillers, graphene, and even phase change composite PCMs made of AlN/paraffin (PA) [77], expanded graphite (EG) [78], and epoxy resin, can be used as additional thermal conductive materials. On top of it, among the available PCMs, the one with the highest thermal conductivity can be chosen for the application. Currently, paraffin wax is utilized since it possesses properties suitable for electrospinning. In the case of the thermoresponsive and shape memory polymers, such as conductive graphene-coated spiky nickel nanoparticles as the conductive filler and a polymer matrix polypropylene (PP) or polyethylene (PE) used as a coating layer on the current collector of the cathode are present; however, the fabrication of separators using these advanced materials by electrospinning technique is not progressed currently [79,80,81].

A novel redox-active separator of [Fe (CN)_6_]^4−^ doped polypyrrole (PPy) composite nanofibers [82] was fabricated via an electrospinning technique followed by in situ polymerization. The working of the system with this novel redox-active separator is illustrated in Figure 7II. This separator composite involves battery reaction and, thereby, enhances the capacity of LIB also. These separators can achieve a high energy density of 56.1%. The separator can maintain the dimension up to 160 °C and is thermally stable up to 289 °C. The improvement in structural properties is due to the nanofibrous structure, and the enhancement in redox activity is due to doping.

## 4. Advancements in Electrospun Anode Materials

The anode is the negative electrode of the electrochemical cell. There are three mechanisms of energy storage for the anode. The working principle of anode materials and its advantages and disadvantages are given in Table 2.

### 4.1. Anode Exhibiting Energy Storage by Insertion Mechanism

Graphite is the most commercially used anode for LIB batteries; however, for more advanced applications, features, such as fast charging, are not achievable with the current graphite-based anode, and the cyclic stability and coulombic efficiency must be increased to power large electrochemical energy storage stations. The graphite materials’ inherent drawbacks are low theoretical capacity and Li dendrite formation caused by intercalation and deintercalation through the inter basal plane in anisotropic graphite microstructure. To match the energy requirement for greater energy storage systems, such as electric vehicles, the intrinsic capacity of the most used anode; graphite (372 mAh/g) should be enhanced. There is always increasing research on graphite material to improve the rate capability, specific capacity, cycle stability, and safety of graphite anodes. However, it is challenging to improve the capacity since it is feasible by creating more space between the layers of graphite [83].

In the case of the materials exhibiting the insertion mechanism, the shortcoming of theoretical capacity can be enhanced by providing additional room for the Li ions; this can be achieved by increasing the surface-to-volume of the material by including carbon-based nanomaterials such as the carbon nanofiber (CNF) [84], carbon nanotube (CNT) [85]. In addition, if the Carbon material added is hollow or porous, it will lead to more Li-ion space, hence leading to increased interaction between the electrode and the electrolyte. Recently, an anode exhibiting an insertion mechanism was synthesized using precursors, such as polyacrylonitrile and preasphaltene; the use of presashaltene is novel since it is obtained from coal liquefication residue (CLR), which is an economical and sustainable source [86]. This precursor was fabricated into a nanofiber non-woven fabric. It was noted that electrospinning of non-woven fiber is the best method to utilize the CLR. Owing to the unique structure of the PA-based CF non-woven fabrics (PACF), which has a short diffusion path, disorder, and 3D interconnected conductive structure.

Titanium oxide-based material is also worthy of anode material; TiO_2_ and Li_4_Ti_5_O_12_ are used as they have excellent working potential and can suppress the SEI formation and the growth of lithium dendrites as well [87]. The TiO_2_ has a great specific capacity, and the spinel oxide structure is a zero-strain material; thus, both have distinct properties which are beneficial in its way. As such, excellent cycling stability and high-rate capability have been achieved with the design of nanofiber electrodes [88,89], and full cells based on the nanofiber electrodes exhibited excellent electrochemical properties. These TiO_2_-based anode materials’ shortcomings on low kinetics are solved by fabricating a one-dimensional nanofiber; this designed nanofiber structure through an electrospinning process exhibits high-rate capability and cyclic stability due to the structure. Further, the electrical conductivity is improved by forming a composite with the addition of conductive materials and through doping as well [90]. Tantalum-doped TiO_2_ was fabricated by electrospinning [91]. Tantalum is used as the dopant because it lowers the diffusion barrier, improves electrical conductivity by shifting the conduction band, and enhances specific surface area by phase transformation. Therefore, the Ta-doped TiO_2_/C nanofibers exhibit good electrochemical performance when made into an anode for LIB and KIB (Potassium Ion Batteries). The specific capacity of the constructed LIB with 5% rutile Ta doping was measured to be 399 mAh g^−1^ at 2 A g^−1^.

### 4.2. Anode Exhibiting Energy Storage by Conversion Mechanism

**Figure 8:**
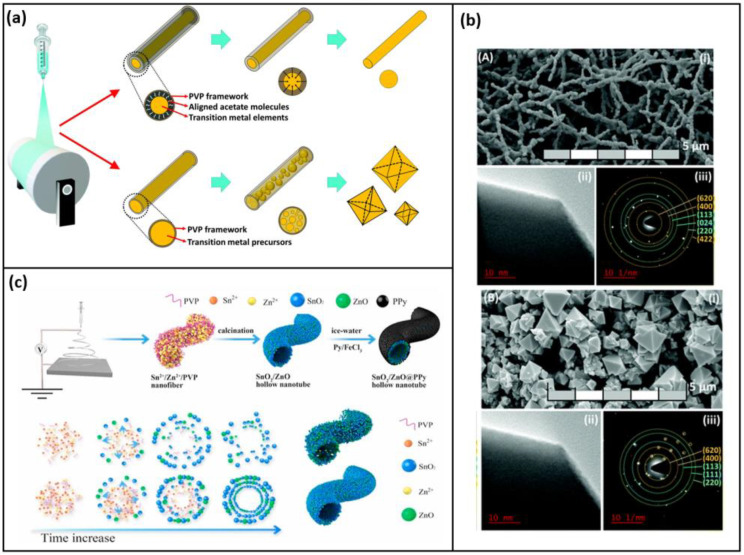
(**a**) Aligned acetate molecules assisted nanostructure formation. (reused with copyrights from Ref. [92] 2022, Elsevier) (**b**) (i) FESEM, (ii) HRTEM, (iii) SAED of the (A) NCM Nanofiber and (B) NCM Octahedron with instruments FESEM, JOEL, JSM-7800F, USA, operating at 30 kV, a TECNAI G2 20 TWIN, FEI-USA respectively (reused with copyrights from Ref. [92] 2022, Elsevier) (**c**) Illustration of fabrication of SnO_2_/ZnO@PPy.s (reused with copyrights from Ref. [93] 2021, Elsevier).

To further improve the capacity, anode material with greater storage capacity is explored, such as silicon, iron oxide, tin peroxide, and cupric oxide. Transition metal oxides possess properties, such as high specific capacity, inexpensiveness, and non-toxicity, which make them a potential candidate for the anode in LIB. These anodes were initially fabricated via the solvothermal and hydrothermal methods techniques forming a 3-D structure, such as core-shell nanospheres, which possess good thermodynamic stability, but pulverization of the active material takes place during the lithiation and de-lithiation cycles, in order to overcome the shortcoming the recent advancement focuses on fabricating a 1-Dimensional structure (Nanofiber, Nanorod, Nano Tubes); thus, the diffusion length is shorter, and movement of Li ions and electrons occurs faster. A quasi-anisotropic nano octahedron as well as nanofiber with nickel–cobalt–manganese oxide composite (NCM), which contains two ternary phases, was fabricated by Ling et al. [92]. To obtain the octahedron structure, the conductivity of the precursor polymer solution is altered. The added Ni helps in the formation of crystalline structure, and the specific capacity increases proportionally with the increase in the Ni Content. The methods and process parameters to fabricate the nanofiber and the nano octahedral structure are similar; the precursor solution is also the same, but in the case of nano octahedral structure, the metal salt precursors have smaller ion sizes than those of the acetate molecules, which is preferred so that the conductivity is enhanced favoring the octahedral structure formation, as shown in Figure 8a,b. The nano-octahedron possesses better properties when compared to the nanofiber due to the synergistic effect.

A 1-Dimensional Transitional metal oxide along with a spinel phase was synthesized by Wang et al. [94], who fabricated MnCo_2_O_4_ (theoretical capacity 906 mAh g^−1^) hollow nanotubes where all the nanoparticles were well connected. This structure was responsible for the outstanding property of the material as it gave several active sites accelerating the rate of transportation and breakage of active material is mitigated. Since the fabrication technique is not tedious, this material finds application as an anode in LIB [95,96,97,98]. For high-performance lithium storage devices, ternary oxides are recommended as it has several electrochemically active components and greater theoretical specific capacity. When the ternary nickel cobaltite was considered, the capacity faded due to pulverization; thus, carbon materials are utilized as it resists the volume expansion and increases conductivity as well. By electrospinning followed by annealing, novel porous NiCoO_2_ nanofibers were fabricated by Wang et al. [99], and the rate of diffusion was fast as the lithium storage sites remained to expand after a continuous cyclic lithiation and delithiation cycle. Thereby the self-assembled nanoparticle exhibited superior electrochemical performance providing an excellent reversible lithium storage capacity (discharge capacity) [100].

In work by J. Zhang et al. [93], Polyvinylpyrrolidone (PVP) of two different molecular weights (Mw = 58,000 g mol^−1^ and 1,300,000 g mol^−1^) was combined with a precursor solution to fabricate a hollow nanotube as shown in Figure 8c, whose morphology changes due to the variation in the molecular weight of the conductive polymer added; higher molecular weight PVP showed higher reversible capacity because of its denser and more stable structure. The fiber synthesized from PVP of higher molecular forms a thinner fiber with a denser packed structure (SnO_2_/ZnO–H), while that of the lower molecular weight (SnO_2_/ZnO–L) exhibits a comparatively loose structure with the macro void, and the fiber is thick. In addition, to enhance the stability and conductivity using in situ polymerization in an ice water bath, the nanotubes fabricated were coated with Polypyrrole (PPy). Among the nanotubes fabricated, the SnO_2_/ZnO–H exhibits better performance owing to its structure. In general, this material has good potential in the application of LIB as it exhibits a discharge capacity of 626.1 mAh g^−1^ of 0.2 C after 100 cycles [101,102,103,104]. The progress in electro-spun anodes exhibiting conversion mechanism is discussed, and the electrochemical properties are listed in Table 3.

### 4.3. Anode Exhibiting Energy Storage by Alloying Mechanism

High energy density battery is achieved by materials storing energy by alloying reaction of energy storage. The Li–Sn alloying exhibits good electrochemical performance; thus, Sn was incorporated into carbon materials and studied. However, the pulverization limits the application; thereby, voids were introduced in the electrospun fibers by fabricating coaxial bamboo-like composite of Sn@C nanoparticles in hollow CNF [106], a porous multi-channeled fiber with Sn nanoparticles reinforced by using single nozzle electrospinning, where PMMA poly(methyl methacrylate) aids the pore formation [28]. In another procedure, Poly Styrene was used to generate voids in the porous CNF incorporated with rattle-like tin nanoparticles [107]. Thus, through the electrospinning process, void engineering is also feasible, thereby providing beneficial electrochemical properties.

**Figure 9:**
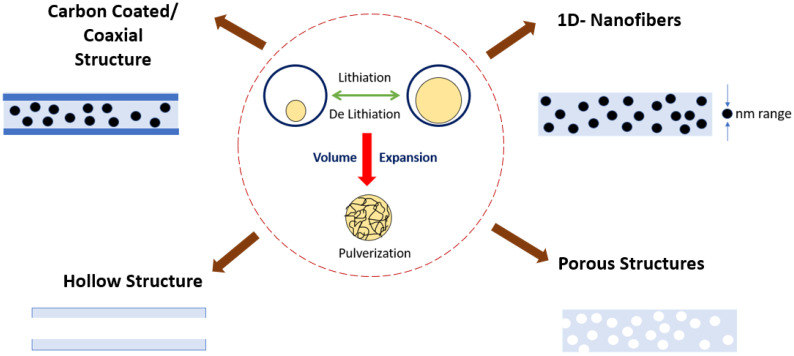
Strategies to overcome the pulverization of active material.

The most researched material exhibiting alloying-type energy storage is silicon since it can be used commercially, as it has an excellent specific capacity of (4212 mAh/g) and is economical as well. However, it suffers a volume expansion of 400% during the lithiation and delithiation process, leading to the pulverization of the active material [108]. Thereby improvements are included in the electrospinning process; the pulverization can be tackled by using (i) Si of nano dimension since the breakage is not expected to occur as the size of Si is already small, (ii) providing voids that can accommodate the volume change by using porous or hollow structures, (iii) creation of void spaces which can accommodate the volume change by carbon coating [109,110]. The carbon coating will be able to enhance the conductivity and block the direct contact of the electrolyte with the electrode. The schematic of techniques to overcome pulverization is shown in Figure 9. The oxide of Silicon also can be used to tackle the volume expansion, and it can maintain long-term stability because, during the alloying reaction, Li_2_O and Li_4_SiO_4_ from the SiO_2_ phase are formed [111], which reduces the electrical conductivity and mobility of the Li-ion leading to loss of irreversible capacity as well. When the mass ratio of the pore-forming element in the precursor solution is altered, the pore structure present in the electro-spun nanofiber can be optimized; thus, Si nanoparticles embedded porous CNF composite nanofiber was obtained, which exhibited superior performance owing to the porous structure. In a work by Tian et al. [112], they optimized the pore-forming with Polyethylene glycol (PEG) since it influences the pore formation. The cycling and rate capacities have significantly improved due to the formation of porous structures.

Other anode materials, such as phosphorous and germanium, exhibit phenomenal theoretical capacities of 2595 mAh/g and 1626 mAh/g for Li_3_P [113] and Li_4.4_Ge [114] phases, respectively. Phosphides can store energy by a dual electrochemical process when combined with metals, such as Sn, Sb, Bi, and Pb, out of which tin phosphides exhibit a greater capacity; thus, by combining electrospinning and solid-state synthesis, nano metal phosphides (SnP_0.94_) were combined with CNF to form a composite anode material [115]. Phosphorous has gained attention nowadays; apart from its high lithium storage capacity, it is available abundantly and can be recycled easily. There are two allotropes of Phosphorus, namely, black and red. Red is utilized as an anode instead of black because processing red needs a higher temperature.

### 4.4. Advanced Electro-Spun Anode Material

#### 4.4.1. Composite

**Figure 10:**
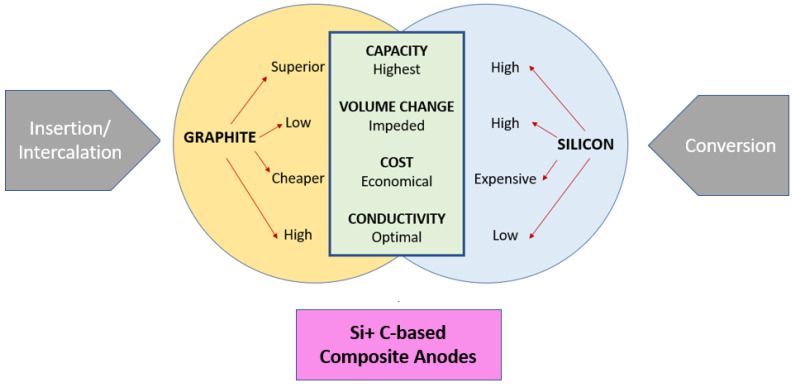
Composite energy storage mechanism.

These materials with conversion-type storage mechanisms suffer greater volume change; thereby, the active anode material is encapsulated in a carbon-based structure forming composites. These composites are of greater interest as they have properties between the materials exhibiting the insertion and intercalation storage mechanism, as shown in Figure 10. These composites are fabricated via the electrospinning technique, and various structures can be achieved, which is advantageous for the performance as anode material. More active sites are generated because of doping using heteroatoms, such as B, S, and N, which enhance the capacity of lithium storage. Defects that cause the carbon structure to become disordered are incorporated, which makes this easier. Carbon’s electrical structure is altered by doping, which increases the number of electrochemically active sites that may be used to store lithium. Heteroatoms in CNF cause an expansion of interlayer distance, generating additional active sites and improving electrical conductivity.

To demonstrate the utilization of transition metals as an anode, to circumvent the low columbic efficiency and reversible capacity hindrance challenges presented by the N-doped carbon fiber alone, the author developed a composite nanofiber of TiO_2_@C/N [116], using a flexible film consisting of TiO_2_ nanoparticles [117] that were incorporated in nitrogen-doped carbon nanofiber. Even without the addition of a conductive binder or other chemicals, the nanofiber developed serves as the anode of the battery. Both the fiber’s performance in the electrochemical system and the nanofibers’ favorable shape for the purpose of electron conductivity have been confirmed. The performance of the charge cycle and the discharge were consistently attributed to the substantial lithium storage capacity of the electro-spun composite. The fibers also have a good rating capacity, and the electrical impedance also diminishes as the number of cycles climbs [118,119,120].

**Figure 11:**
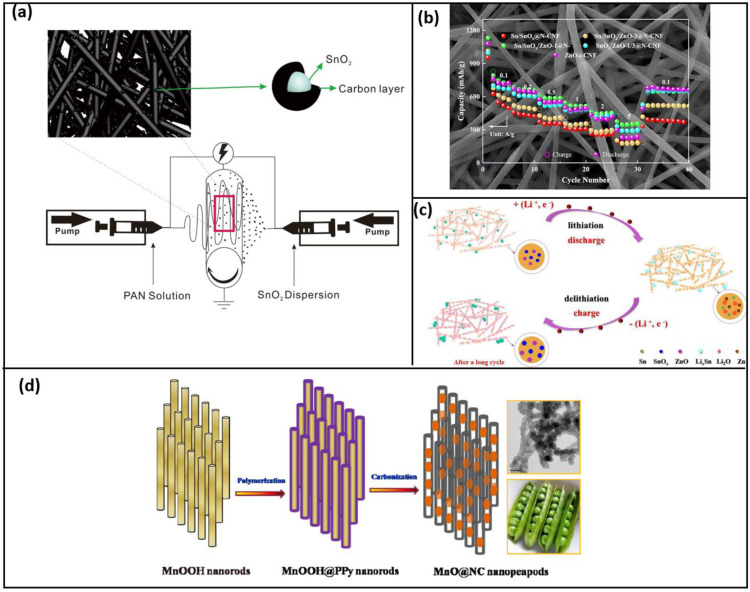
(**a**) Schematic of synthesis of carbon–coated SnO_2_@carbon nanofibers by synchronized electrospinning and electrospraying (reused with copyrights from Ref. [131] 2019, Elsevier) (**b**) Sn-coated SnO*_x_*/ZnO@N-CNF composites exhibiting superior properties (reused with copyrights from Ref. [125] 2020, Elsevier) (**c**) The schematic of energy storage mechanism of Sn/SnO*_x_*/ZnO@N-CNFs (reused with copyrights from Ref. [125] 2020, Elsevier) (**d**) Fabrication of nano peapods of N-doped carbon-coated MnO nanostructures (reused with copyrights from Ref. [128] 2013, Royal society of chemistry).

Electrospinning and electro-spraying were combined to develop a composite made of carbon nanofibers, along with carbon-coated SnO_2_ clustering, and SnO_2_ clusters adhered over the CNF [121] to achieve a stable hybrid structure that boosts electron transfer and ionic diffusion as shown in Figure 11a. Lithium transport was, therefore, seen to be enhanced when SnO_2_ was coated outside, which boosted battery efficiency [122,123,124]. A pseudo capacitor behavior is brought in the LIB by the synergistic effect when SnO_2_ (high theoretical capacity) and ZnO (high co-efficient of diffusion of Li ion) composite is coated with Sn and N-doped CNF hybrid material. In the study by L. Shang et al. [125], the mass ratio of SnO_2_ and ZnO precursor varied as 1:1, 3:1, and 1:3, along with the reference where the single precursor alone was only utilized. Among the different mass ratios, the material synthesized by equal proportions of both the precursor [Sn (Ac)_4_ for SnO_2_ and Zn (Ac)_2_ for ZnO] exhibited a comprehensive performance as LIB anode material.

Wang et al. [126] overlayed nitrogen-doped CNF and metal oxide in a hybrid structure to maximize the anode material’s performance. He only used MnO nanostructure in his studies since it had minimal voltage hysteresis and a large theoretical capacity. The lithiation/delithiation process causes significant volume fluctuation and continuous cycles [127]. However, the inclusion of carbon improves conductivity and reduces the structural strain that results. Although the limited elasticity of MnO can cause carbon layers to fracture due to volumetric expansion, a novel nano-peapod structure, Figure 11d, was created to address this shortcoming [128]. This structure consists of an encasing carbon pod and an internal void room that acts as an elastic buffer layer [129,130].

The Li storage performance is attributed to the structure of the fiber, which also generates an efficient pathway of transport for all electrolyte penetration, Li ions, and electrons as well. When nanoparticles of FeCo were added to Nitrogen-doped CNF due to a synergistic effect, outstanding electrochemical properties were obtained [132]. A reversible capacity of 566.5 mAh g^−1^ was recorded at the end of 100 cycles at a current density of 100 mA g^−1^. These fibers could be used to produce batteries without the addition of any conducting or binding materials, and when evenly scattered FeCo nanoparticles restrict volume change and increase structural stability throughout charging and discharging cycles. The addition of FeCo increases the composite material’s Columbic efficiency while simultaneously acting as a catalyst for Li_2_O breakdown [133,134].

With the help of electrospinning and heat treatment, SnO_2_/TiO_2_ ultrafine particles with a molar ratio of 1.5:1 have been combined with carbon nanofiber. Here, SnO_2_ is used since it has a higher theoretical capacity and is easily obtainable [135], and TiO_2_ is used as it also provides greater electrochemical stability, and the significant volume change that occurs during the reaction is shown to be diminished to a greater extent, improving overall performance. The results show that the composite was successfully synthesized because it has a narrower Li-ion diffusion path, which enhances the kinetics of the electrochemical reactions, as shown in Figure 11b,c [136,137,138,139].

LIB is also used in wearable electronic devices; thus, it requires a flexible electrode that does not damage the active material present when it is bent; thereby flexible electrodes which do not compromise the electrochemical performance are areas of increasing demand; thus, a porous Sb_2_S_3_/TiO_2_/C nanofiber membrane was fabricated using a titanate coupling agent (titanium (IV) isopropoxide (TTIP)) as a precursor as it has the capacity to entangle with the polymer and, thus, bridging between the polymer chose and transition metal ion, providing an improved thermotolerant mechanical property [140]. Antimony sulfide (Sb_2_S_3_) was considered a potential material for the anode due to its low cost, higher theoretical capacity, and environmental friendliness. Using electrospinning and hydrothermal reaction to create a porous nanofiber membrane that was employed as a free-standing anode and has good cyclic stability and better capacity as well, the porous structure forbids a greater volume expansion. The fourfold folding of the fiber did not cause it to wrinkle, demonstrating mechanical flexibility [136,139,141].

In addition to the generally utilized methods, such as the restriction of particle size and dispersion of the active material in a conductive matrix, L. Jiao demonstrated the benefit of the inclusion of an inactive component that can attenuate the volume change. In his research, he employed Sn as the active substance and Co as the inactive substance, and nitrogen, which were doped in CNF. Co was chosen among the available inactive materials, such as Mn, Fe, Cu, Co, and Ni, to help maintain the electrode’s integrity during lithiation and de-lithiation. This metal also raises the usage efficiency of Li ions, given that it is immobile and does not consume Li. Additionally, the synergistic combination of N-doped CNF with the Co metal accelerates the rate of ionic diffusion and, as a result, the electron transport since it is electrically conductive. The energy density of the LIB is boosted since neither a binder nor a current collector is involved. The manufactured fiber with a diameter of 100 nm functioned well.

Metal and metal oxide are possible options to fulfill the growing need for LIB; nevertheless, even after frequent cycles of lithiation and delithiation, these materials also fracture. J. Li et al. reported on the process of manufacturing a free-standing and flexible membrane made of Sn@C [142] nanofiber because the carbon matrix is one-dimensional and contains Sn nanoparticles that are enclosed inside the structure. This electro-spun structure has an excessive current density of 10 A g^−1^ and a capacity of 668 mAh g^−1^ at 1 A g^−1^. The confinement brings such extraordinary qualities. The energy density is enhanced by omitting binders or collectors, which may be made without the need for additional slurry coating. The carbon coating can absorb a sizable volume change without causing the active material to be destroyed, which would otherwise result in fracture during the lithiation and delithiation cycle. As a result, it provides the potential for LIB industry growth in the anode [142,143].

A nanostructured carbon-based anode material with red phosphorous was fabricated by Liberale et al. [144]. This structure helps to overcome the limitation of phosphorous (high volume expansion, low electronic conductivity) as the C/P bonds allow to shorten Li^+^ diffusion path and stabilize P during cycling [145,146]. The amorphous composite fabricated by electrospinning the CNF followed by drop casting the phosphorus to obtain uniform dispersion possessed excellent electrochemical properties as well [147,148,149].

#### 4.4.2. MOF Derived

To overcome the volume change, a new material was introduced, namely, MOF-derived metal oxides [150,151]. These materials have the capacity to adjust to the volume change; thus, it overcomes the existing issue faced by all materials discussed earlier [152,153]. Apart from volume change, the MOF–derived material has more structural stability and improved electrochemical performance as this porous metal oxide decreases the diffusion pathway and provides more active sites. The distinctive structure and morphology contribute to the outstanding electrochemical performance because the MOF precursor, during the peroxidation and pyrolysis process, forms more Li-ion reservoirs as nitrogen-doped or carbon-coated metal oxide is generated during the fabrication process.

Z. Li et al. fabricated Fe_2_O_3_@ Polyacrylonitrile (PAN) and ZnO@PAN [154] composite nanofibers using an electrospinning technique followed by subsequent pyrolysis of the precursor film, they exhibited a specific capacity of 1571.4 and 1053.8 mAh g^−1^ at 50 mA g^−1^, respectively, and the reversible capacity of the respective composites after 500 cycles at 1000 mA g^−1^ were 506.6 and 455.4 mAh g^−1^, which is primarily due to the interconnection of the CNF with the metal oxides [155]. Carbon nanofibers embedded with cobalt were fabricated by Y. Liang et al. [156] via pyrolysis of electro-spun fiber in a nitrogen atmosphere. This spindle-shaped nanofiber formed after mild oxidation exhibited superior properties due to the synergistic effect between the carbon shells and the Co present. This Co_3_O_4_@CNFs anode discharge specific capacity of 1404 mAh g^−1^ and 500 mAh g^−1^ after 100 cycles and 100 mA g^−1^ current density and after 500 cycles at 2000 mA g^−1^.

**Figure 12:**
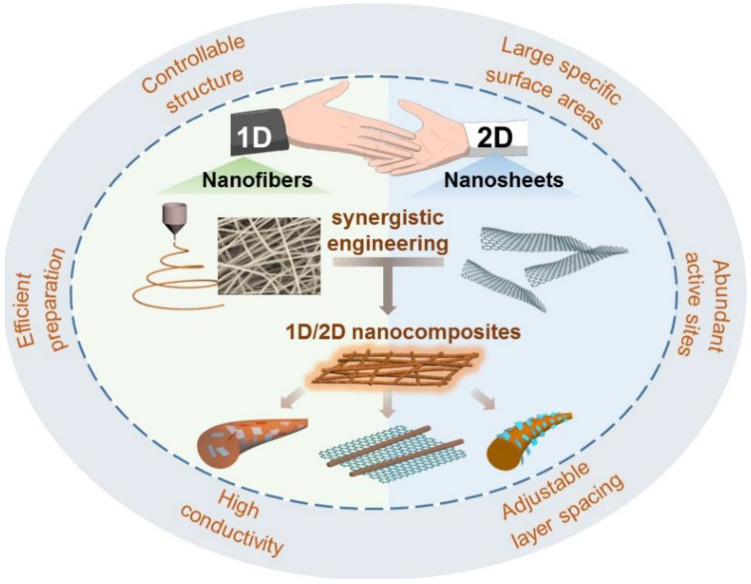
Hybrid Nanocomposites exhibiting outstanding properties brought by Synergistic engineering (reused with copyrights from Ref. [157] 2020, Elsevier).

Although the 2D nanofiber sheets do not share the mechanical and thermal conductivity shortcomings of the electro-spun 1D nanofibers, they certainly possess their own downsides. As a result, a hybrid structure, as shown in Figure 12, with both 1D and 2D nanomaterials, was created, and the synergistic engineering was able to offer remarkable electrochemical capabilities. Electrospinning may be used gradually to create a hybrid structure, offering a new development path. The hybrid structure is also made by techniques such as physical blending or in situ growth [157]. A consolidated summary of the advanced electrospun anode materials is provided in Table 4.

#### 4.4.3. Eco-Friendly, Bio-Derived Nanofibers

The future aims to make this electrospinning a much more sustainable process, which can be achieved by the following: (i) making the electrospinning process environmentally friendly by eliminating the possibility of any hazards, such as adopting needless electrospinning techniques, optimizing the process by making it energy-efficient by powering the equipment with rechargeable batteries, which can also be reused to harness its secondary lifetime; (ii) using precursors, which can be replaced with alternative nontoxic chemicals, precursors derived from biomass. The carbon, which is used widely for the electrode, can be derived from bio sources [159,160], such as coffee ground waste [161], shrimp waste [162], marine chitin [163,164], and cellulose [163,165,166].

The electrode is a crucial part that significantly affects the LIB’s overall performance. Conventional graphite electrodes contain volatile N-methyl-pyrrolidone (NMP), which is employed as a solvent, and poisonous polyvinylidene fluoride (PVDF), which serves as a binder. As a result, K. Xu et al. created a unique energy storage technique employing an affordable, eco-friendly carbon electrode material that also has high electrochemical properties. They developed a bio-nitrogen (N)-doped composite carbon nanocomposite mat using chitosan and natural cellulose [24]. The chitosan-based mat was first manufactured via the pyrolysis method, but because of its poor porosity and resolvability, additional fabrication techniques, such as hydrothermal carbonization and direct carbonization, were also employed. Later, with cellulose and chitosan mass ratios of 10:0, 7:3, 5:5, 3:7, and 0:10, the electrospinning technique was used in the single and coaxial nozzle to prepare a porous carbon with good specific surface area and interconnectivity. Better performance was recorded in a mass ratio of 5:5, which exhibited a high specific capacity of 327 mAh g^−1^ at 100 mA g^−1^, good rate performance with a specific capacity of 399 mAh g^−1^ at 30 mA g^−1^, and 210 mA g^−1^ at 1000 mA g^−1^; the stability after 300 cycles was also commendable [158,163,167].

The electrospinning process can be made more energy-efficient by adopting the following strategies: (i) use of direct-current electrospinning instead of traditional high voltage electrospinning as a comparatively lesser voltage is used making the process energy efficient [168,169]; (ii) powering the electrospinning equipment using renewable energy resources, such as solar panels and wind turbines, which can reduce the carbon footprint of the electrospinning process [170,171]; (iii) usage of renewable or biodegradable materials to prepare the fibers as these materials can be broken down in the environment, reducing the amount of energy needed for disposal [172,173]; (iv) to achieve appropriate end-of-life disposal of the electrospun fibers by recycling or composting the fibers as it can reduce the amount of energy required for disposal; (v) reusing the syringe after flushing the polymer melt left after processing using the appropriate solvent [174]; (vi) optimize the process by reducing the waste generated or recycling excess material that is not used in the fiber-formation process [175].

## 5. Conclusions

In conclusion, electrodes and separators are vital components of rechargeable batteries, and with the increasing demand for such batteries, there is a need to develop electrode materials that can provide optimal performance. The electrospinning technique has emerged as a versatile method for fabricating one-dimensional structures. This review paper has highlighted the most recent advancements in electrospun nanofiber fabrication materials and methods. Several modern electrospinning techniques have been used to produce nanofibers with excellent properties, including direction-oriented network non-woven mats, flexible nanofiber, porous nanofiber, encapsulated nanofiber, composite nanofiber, and doped nanofibers. The electrospinning technique has enabled the creation of porous nanofibers with reduced volume expansion and increased electrochemical performance, demonstrating its versatility. Furthermore, ongoing efforts aim to make this process more sustainable. The gradual application of electrospinning can result in hybrid structures that have excellent properties and open up new development routes. In situ growth or physical blending can create hybrid electro-spun structures for future battery components.

## Figures and Tables

**Table 1:** Recent Electro spun Cathode Materials used in LIB.

Cathode Material	Capacity (mAh g^−1^)	Nanofiber Diameter (nm)	Cyclic Stability	Remark	Reference
Fork-like electro-spun K_2_V_8_O_21_	200.2	100	96.52% after 100 cycles	Layer-by-layer nanostructure morphology improves diffusion and accommodates volume change, as the structure provides more contact area	[25]
Ternary (Li_2_CoTi_3_O_8_/TiO_2_) nanofiber	82 at 0.1 C	430	83% after 25 Cycles.	The ternary phase has lower capacity.	[41]
Spinel-layered Li_2_MnTiO_4+z_	210 at 0.1 C	175	95.3% after 100 Cycles.	The spinel structure increases the capacity but reduces the average voltage	[42]
N-Doped C Nanofibers with Uniform Embedding of Mn Doped MFe_1−x_Mn_x_PO_4_ (M = Li, Na)	169.9 at 0.1 C	250	160 after 200 cycles	e lattice distorts after Mn-doping without altering the original crystalline structure, increasing conductivity, and enhancing the efficiency of Li+/Na+ diffusion	[24]
3D LiFePO_4_@rGO/carbon nanofibers	167.1 at 0.5 C		98.9% after 200 cycles	r GO provides effective electron transfer path, CNF prevents accumulation and agglomeration during pyrolysis process	[47]
Li_3_V_2_(PO_4_)_3_ nano cubes/carbon nanofibers	143.6 at 5 C	500	Almost 100%	IL assisted structure directing electrospinning helps to form LVP nano cube.	[52]
Electro-spun LiNi_0.5_Co_0.2_Mn_0.3_O_2_ with polymerization	143.2 at 0.1 C	440	74% after 80 cycles.	Interface engineering was feasible by electrospinning followed by polymerization.	[53]
(010)-oriented LiFePO_4_ nanocrystals/carbon nano fiber hybrid network	152 at 0.5 C		98.2% after 500 Cycles	Direction oriented Nanofibers were fabricated using electrospinning technique.	[23]
Carbon-coated electro-spun V_2_O_5_ nanofibers	146 to 161 for carbon coated. 164 to 179 Uncoated	100–200	Carbon coated VNF 61.13% uncoated VNF (43.85%)	Electrospinning can be used to fabricate cathode for photo responsive batteries	[54]

**Table 2:** Anode working mechanism.

Mechanism	Working	Materials	Advantage	Disadvantage
Insertion or Intercalation	The Li ions are inserted and removed from the anode material reversibly without significant microstructural change	Graphite and Li_4_Ti_5_O_12_	An extended life because of structural stability and smaller volume change during the electrochemical Reaction.	Theoretical capacity is very low
Conversion metal reaction-based metal anode	The storage of lithium is based on altering metallic bond	Fe_2_O_3_, Fe_3_O_4_, Co_3_O_4_, NiO, and MnO	Higher specific capacity	Greater volume change, poor kinetics, and great redox potential hysteresis
Alloying anode material	By rupturing the inter-atomic interactions of the host component, lithium atoms combine with the host metallic phase to produce an alloy	Silicon, germanium, antimony	Improved specific capacity and low working potential	The growth of solid electrolyte interphase (SEI) growth, loss of electrical contact, and pulverization due to greater volume expansion and contraction
Plating anode material	Utilization of inactive lithium surface for deposition which can be later stripped by oxidation reaction.	Electrospun Nanofiber mat	Volume change is minimized due to the porous structure, current densities are reduced by a large specific surface area.	The contact between the nanofiber mat and lithium

**Table 3:** Electrochemical properties of advanced anode material exhibiting conversion storage mechanism.

Material	Specific Capacity	Nanofiber Diameter (nm)	Capacity Retention	Initial Capacity Loss	Rate Performance	Reference
NCM nano-octahedron	1183.5 mAh g^−1^ at 100 mA g^−1^	800	78.9% after 500 cycles at 1000 mA g^−1^	37.4%		[92]
NCM nanofiber	1074.8 mAh g^−1^ at 100 mA g^−1^	800	24.1% after 500 cycles at 1000 mA g^−1^	38.7%		[92]
MnCo_2_O_4_ nanotubes	701.4 mAh g^−1^ at 500 mA g^−1^ after 320 cycles.	450			400.4 mAh g^−1^ at 1 A g^−1^	[94]
Porous NiCoO_2_ nanofibers	945 mAh g^−1^ at 100 mA g^−1^ after 140 cycles	800			523 mAh g^−1^ at 2000 mA g^−1^	[99]
SnO_2_/ZnO@PPy hollow nanotubes	626.1 mAh g^−1^ at 0.2 C of 100 cycles (Discharge Capacity)	303				[105]

**Table 4:** Electrochemical properties of advanced anode material.

Material	Specific Capacity	Nanofiber Diameter (nm)	Coulombic Efficiency	Discharge Capacity	Reference
TiO_2_@C/N composite NF	388 mAh g^−1^ after 400 cycles.	800		563 mAh g^−1^ at 0.1 A g^−1^	[116]
Carbon-coated SnO_2_@carbon nanofibers	500 mAh g^−1^ after 50 cycles	400	63.24%.	1425 mAh g^−1^ at 100 mA g^−1^	[121]
Sn particle-coated composite of SnO_x_/ZnO and N-doped CNF	1131 mAh/g	350	73.2% at 0.5 A/g	588.7 mAh/g after 100 cycles at 0.5 A/g (Reversible capacity)	[125]
Nitrogen-doped carbon coated MnO nano peapods	775.4 mAh g^−1^ at 100 mA g^−1^	125		rate capacity of 559.7 mAh g^−1^ at 1000 mA g^−1^ s	[126]
FeCo nanoparticles encapsulated in N-doped carbon nanofibers	566.5 mAh g^−1^ at 100 mA hg^−1^ after 100 cycles. (Reversible Capacity)	130	99.6%		[132]
Ultra-fine SnO_2_/TiO_2_ particles into carbon nanofibers	766.1 mAh/g.	600	71%, and after 200 cycles	1494.8 mAh/g Charge-1061.2 mAh/g,	[135]
Sb_2_S_3_/TiO2/C nanofiber				261.6 mAh g^−1^, 100 cycles, 50 mA g^−1^.	
Co/Co_3_SnC_0.7_@N-CNFs	320 mAh g^−1^ at 500 mA g^−1^ even after 900 cycles.				
Red Phosphorous decorated carbon anode.					[144]
MOF derived					
Fe_2_O_3_@ Polyacrylonitrile (PAN)	1571.4 mAh g^−1^				[154]
ZnO@PAN composite nanofibers	1053.8 mAh g^−1^				
Co_3_O_4_@CNFs	1404 mAh g^−1^ (100 cycles and 100 mA g^−1^)	100			[156]
Bio-nitrogen (N)-doped composite carbon nanocomposites mat using chitosan and natural cellulose	327 mAh g^−1^ at 100 mA g^−1^	316			[158]

## Data Availability

Data will be made available upon request.
